# Glycemic control of diabetes patients under continuous rocket attacks

**DOI:** 10.1186/s40696-016-0011-x

**Published:** 2016-01-12

**Authors:** Varda Soskolne, Rachel Dekel, Shlomo Vinker

**Affiliations:** 1 The Louis and Gabi Weisfeld School of Social Work, Bar Ilan University, 52900 Ramat Gan, Israel; 2 Department of Family Medicine, Sackler School of Medicine, Tel Aviv University, Tel Aviv, Israel

**Keywords:** Type 2 diabetes mellitus, Trauma, Rocket attacks, Glycemic control, Risk factors for complications, Israel

## Abstract

**Background:**

Evidence regarding the detrimental effects of exposure to stress on glycemic control among diabetes patients has mainly focused on personal life events or acute trauma. However, the effects of continuous exposure to extreme stress on type 2 diabetes patients have rarely been studied. The aim of the current study was to examine the association of continuous exposure to rocket attacks with glycemic control and with risk factors for diabetes complications among civilian type 2 diabetes patients. We focus on patients residing in the Western Negev in the south of Israel that has been subjected to rocket attacks fired from Gaza since the end of 2001.

**Methods:**

A two-arm retrospective cohort study of type 2 diabetes patients, aged 35–70 years, residing in a region with chronic exposure to rocket attacks (N = 1697) and in a non-exposed comparison region in Israel (N = 3000). Data were retrieved from the Health Maintenance Organization (HMO)’s database for four time periods representing exposure: chronic—2008; elevated—2009 (post’Cast Lead’ operation); return to chronic—2010, 2011. Data included socio-demographic variables, HbA_1c_, BMI, LDL cholesterol, blood pressure. General Linear Models (GLM) were used for analysis.

**Results:**

For HbA_1c_, the model yielded a significant main effect for time, a borderline significance main effect for region, and a significant time by region interaction: no differences in HbA_1c_ levels between the regions in 2008 and 2009, followed by significant differences between the regions in 2010 and 2011 when HbA_1c_ continued to increase in the exposed region but decreased in the comparison region. Regarding risk factors, a significant main effect for time for LDL cholesterol only, and significant main effects for region were found in all factors: BMI and LDL cholesterol were higher in the exposed than in the comparison region, but blood pressure values were lower.

**Conclusions:**

Continuous exposure to rocket attacks is associated with glycemic control and risk factors in a complex pattern. These preliminary findings require further studies of diverse types of civilian exposure to continuous extreme stress.

## Background

A growing body of research has indicated that exposure to stressors, such as life events or chronic difficulties, has detrimental effects on medical state of people living with a chronic disease, among them diabetes patients [1]. Evidence on the impact of severe events, acute or long-term traumatic experiences, on glycemic control is more limited and inconsistent. Several studies showed that surviving an acute event, such as floods [2] an earthquake [3, 4] or war [5] led to a significant increase in glycated haemoglobin (HbA_1c_) levels, followed by a gradual decline to pre-event levels, while another study found non-significant changes [6]. Others indicated that higher levels of exposure to the traumatic event were associated with elevated HbA_1c_ levels [7]. These studies suffered from methodological problems such as small sample size or recruitment of a non-representative sample of patients from a single medical center. Moreover, these studies examined a single event and not continuous exposure to traumatic experiences. In an attempt to expand the scientific knowledge on the impact of severe long-term exposure on medical state of type 2 diabetes patients and to overcome methodological limitations of previous research, our study examined the traumatic experience of exposure to terror-related events. Such events have increased in the past two decades affecting civilian populations in many parts of the world. Yet, to the best of our knowledge, their impact on clinical state of people living with chronic illness has not been studied. The present study examined the association between continuous exposure to rocket attacks and clinical indicators among type 2 diabetes patients: glycemic control and major risk factors for diabetes complications—obesity, lipid level and hypertension. We focus on residents of the Western Negev in Israel, a region that has been subjected to continuous rocket attacks fired from Gaza since the end of 2001.

## Methods

### Study design

In this two-arm cohort study, two geographic regions in Israel were selected: (a) Chronic exposure—a town (Sderot) and rural villages within a 20-kilometer radius surrounding the Gaza Strip, which have been subjected to continuous rocket attacks fired from Gaza since the end of 2001, with accelerated frequency in 2007–2008 of 8–9 rockets a day, claiming lives, hundreds of physical casualties, and causing thousands of anxiety attacks [8], (hereafter exposed region). (b) No exposure—towns and villages of similar demographic background from Israel Central region, not exposed to rocket attacks (comparison region). We carefully selected towns of the same rank in the socio-economic index as Sderot, or only one rank lower or higher, south of but not from Tel-Aviv metropolitan area, as well as villages of similar size. Four time-periods representing different levels of exposure to attacks in the exposed region were examined. Time 1: continuous, chronic exposure (2008); Time 2: elevated (2009)—continuous exposure combined with acute exposure during “Cast Lead” operation, last days of 12/2008 through January 2009, when about 660 rockets fell mainly in the exposed region, yet reaching further to towns in the South not affected before, and accompanied by wide media coverage [8]; Time 3 and Time 4: return to continuous although decreased and sporadic exposure (2010, 2011).

### Data source and study variables

Data of type 2 diabetes patients, aged 30–70 years, insured by Clalit Health Services (hereafter HMO), residing in the two regions were included. After approval of the study protocol by the HMO’s Ethics Committee, all patients in this age range from the exposed region (N = 1697) and a random sample of 3000 patients from the comparison region were selected from the HMO computerised database. Data on age, gender, socio-economic status (SES), measured by a dichotomous variable (yes vs. no exemption from co-payments, an unspecific indicator of poverty level), and for each time period, HbA_1c_ values, and risk factors—LDL cholesterol, BMI (kg/m^2^), systolic and diastolic blood pressure were retrieved from the HMO database at the end of 2011. In order to capture the possible reaction to the acute state in early 2009, data for HbA_1c_ were restricted only to those from January–June 2009 (the values closest to January–March 2009); while for the risk factors any annual test result was taken for each year. In most cases, only one value was recorded at each time period.

### Description of the sample

Mean age was 59.5 (8.5), 53 % were men. Patients in the exposed vs. the comparison region were significantly younger [58.8 (9.4) and 59.9 (8.0), respectively, *p* < 0.01], a smaller proportion were men (51 and 55 % respectively, *p* < 0.05). Additionally, a small but significant difference was found between the two regions in SES: a higher proportion of patients (33 %) in the exposed region than those in the comparison region (27 %, *p* < 0.001) were exempt from co-payments.

### Statistical analysis

Descriptive statistics were assessed and bivariate differences between the two regions were tested using *t* test for continuous variables and χ^2^ tests for categorical variables. A series of General Linear Models (GLM) was conducted to examine the effect of region (between group differences) and time periods *(*within group differences) on glycemic control and risk factors and included interaction terms for region with time, controlling for age and sex. *p* value in all the models was set at *p* < 0.05 for statistical significance. Data of laboratory test results for some of the indicators and recorded blood pressure values were missing in the HMO database. This may be a source of selection bias because patients who do not come for regular follow-ups could differ from those who did; yet we found no significant differences by age, gender, SES or region in any of the measures.

## Results

The GLM results are shown in Table 1. For glycemic control, the model yielded a significant main effect for time, and of a borderline significance (*p* = 0.065) main effect for region. Additionally, the model yielded a significant time by region interaction: There were no differences in HbA_1c_ levels between the regions before (2008) or during the acute period (2009), and the levels increased in both regions from 2008 to 2009. However, in the follow-up years 2010, HbA_1c_ level continued to increase in the exposed region but decreased in the comparison region. Examining the source of the interaction revealed significant differences between the regions only in 2010 and 2011, and significant differences within each region between the 2008 and all the other times (*p* < 0.05, after Bonferroni correction). Yet, the effects of time, region and the interaction are minimal (<1 %).

**Table 1:** General Linear Model (GLM) for medical status variables

	Time 1 (2008)		Time 2 (2009)		Time 3 (2010)		Time 4 (2011)			*p* value^a^ (Eta^2^)		
Region	Exposed	Comparison	Exposed	Comparison	Exposed	Comparison	Exposed	Comparison		Time	Region	Time ×region
Measure	Mean (SD)		Mean (SD)		Mean (SD)		Mean (SD)					
HbA1c (%)	7.14 (1.48)	7.12 (1.38)	7.41 (1.57)	7.45 (1.49)	7.52 (1.46)	7.33 (1.38)	7.68 (1.51)		7.34 (1.50)	<0.001 (0.005)	0.065 (0.002)	<0.001 (0.007)
HbA1c (mmol/mol)	54.5	54.3	75.7	57.8	58.7	56.6	60.3		56.7			
*n* ^b^	631	1160										
BMI (kg/m^2^)	30.75 (5.44)	30.09 (5.90)	30.67 (5.58)	30.09 (5.66)	30.58 (5.62)	30.00 (5.64)	30.29 (5.45)		29.80 (5.56)	0.790 (0.000)	0.031 (0.003)	0.433 (0.000)
*n*	742	1279										
LDL Cholesterol (mmol/l)	2.67 (0.88)	2.57 (0.79)	2.53 (0.85)	2.48 (0.80)	2.45 (0.87)	2.38 (0.77)	2.36 (0.85)		2.28 (0.75)	<0.001 (0.002)	0.007 (0.002)	0.254 (0.000)
*n*	1499	2667										
Systolic BP (mmHg)	131.6 (16.37)	134.1 (17.14)	130.7 (15.63)	132.8 (15.50)	131.9 (17.23)	131.5 (15.92)	129.9 (14.57)		130.6 (14.92)	0.267 (0.001)	0.054 (0.002)	0.002 (0.002)
*n*	755	1312										
Diastolic BP (mmHg)	76.34 (8.83)	77.42 (9.34)	75.92 (8.67)	76.54 (8.23)	75.08 (9.42)	75.35 (8.68)	73.72 (9.26)		74.10 (8.34)	0.343 (0.001)	0.007 (0.004)	0.327 (0.001)
*n*	755	1312										

^a^p values are based on F test, controlling for age and gender

^b^ *n* for HbA_1c_ was restricted to those tested January–June 2009. *n* for other measures—any test during the year

The GLM models of risk factors yielded a significant main effect for time only for LDL cholesterol, which improved over the years, and significant main effects for region in all risk factors. Compared to patients in the comparison region, patients in the exposed region had higher BMI and LDL cholesterol levels but lower blood pressure values. Additionally, the model for systolic blood pressure yielded a significant time by region interaction: the levels decreased in the comparison region over time (significant differences between 2008, 2009 and 2011), they fluctuated in the exposed region and were significantly different from those in the comparison region in 2008 and 2009 (*p* < 0.05, after Bonferroni correction).

## Discussion

Our findings demonstrate that exposure to continuous rocket attacks was related to a progressive poor glycemic control, even when the frequency of attacks subsided. Yet glycemic control of patients in the exposed region differs from that of patients residing in a non-exposed region only in the years following an acute stress. Less consistent are the differences in risk factors: while the patients in the exposed region also have higher BMI and LDL cholesterol levels than those shown for the comparison region, their blood pressure levels were lower.

Previous evidence on the effects of stress on glycemic control focused on exposure to acute, natural events [2, 4] or on war stress that affects the total population [6] and relied on small samples [5]. The current study is the first to examine exposure of a civilian population of diabetes patients to continuous threat of intermittent rocket attacks. Its strengths include the incorporation of risk factors in addition to HbA_1c_, a large sample size, of community dwelling patients, a comparison region, and a longer follow-up.

Our analysis demonstrates a complicated pattern of the consequences of continuous exposure and acute attack periods. The interaction of time by region for HbA_1c_ showed that there were no significant differences between the regions in 2008, despite the fact that the exposed area was already subjected to rocket attacks since 2001. This pattern could be explained, in part, by the habituation hypothesis, suggesting that repeated exposure to a stressful event may serve to normalize perceived threats and make the circumstances of unusual events more understandable [9]. Thus, victims become toughened and more resilient to subsequent experiences [10].

Second, while we expected an increase in 2009 in the exposed area, following “Cast Lead” operation, the similar increase in the level of HbA_1c_ in the comparison region, indicates that this stressful time affected patients in other regions via media exposure or personal contacts. Reactions to such indirect exposure are known to be expressed in elevated levels of distress symptoms [11], even reaching the same magnitude of the exposed individuals [12]. Others support our findings that the reactions to indirect exposure are also expressed in an increase of medical problems, such as those found in the US general population following the 9/11 attacks [13]. Once the acute period was over, HbA_1c_ values decreased among the comparison patients while they continued to increase in 2010 and 2011 in patients in the exposed region. One potential explanation may be that habituation may have its limits: the residents in the exposed region were expecting that there would be a quiet period following the military operation but the rocket attacks continued (although more sporadically).

The increased risk for diabetes comorbidities was also expressed in the significantly higher levels of BMI and LDL cholesterol among patients in the exposed region, suggesting that they may have had more difficulties in adherence to healthy life style and/or impaired compliance to medications. Additionally, although systolic and diastolic blood pressure values were lower in patients in the exposed region, the decrease (in systolic BP) over time was smaller than in patient in the comparison region. In view of the absence of findings on changes in these medical factors in previous studies, our findings are preliminary. Further examination is required in order to understand the physiological mechanisms of the effects of the risk factors and in conjunction with HbA_1c_, as part of the neuroendocrine system role in response to stress. One assumption is that responses to acute stressful events that are protective and adaptive in nature differ from those to chronic stress which elicits neurochemical, neuroanatomical and cellular changes that may have deleterious consequences upon higher brain functioning [14].

Our findings suggest that the continuous chronic and acute stress periods of exposure to rocket attacks has complex pattern of consequences for glycemic control: no difference between the regions after several years of exposure (the already chronic state in 2008), but an activation of reaction—poorer glycemic control—after an acute period. However, this pattern should be interpreted in the context of the study limitations. First, no causality can be assumed as we lack of data on glycemic control in the pre-exposure to rocket attacks period and the first years of exposure. Second, although the analyses controlled for demographic differences, it may be that despite our efforts in selecting similar towns and villages (not from a metropolitan area) in the comparison region, differences in the delivery of medical care between central and peripheral regions persisted. Due to our unmatched design we cannot rule out the possibility that patients in the comparison region differed on other important variables unknown to us, such as adherence to diabetes self-management or depression. A third limitation relates to the generalizability of our results. Continuous rocket attacks are a unique type of extreme traumatic stress, and reactions to other types of continuous traumatic situations may be different. Fourth, our reliance on data retrieved from the HMO database restricted our ability to adjust for additional confounders (e.g., robust socioeconomic status measures, the number or intensity of prescribed medications, adherence to medications) and was compounded by missing test results for some of the indicators that, although no selection bias was detected, was subjected to other differences in ways we were unable to measure. Therefore, our preliminary findings should be further examined in studies with a matched case design of patients by age, gender, SES and duration of diabetes, and include a wider array of variables, and different patterns of exposure to chronic extreme stress. They will benefit from inclusion of more representative samples as well as other chronic patients in order to reach better conclusions about long-term effects.

## Conclusions

The current study, although being preliminary, provides data that, to our best knowledge, have not been studied before. These findings have significant implications for clinical practice. Health care professionals need to be aware of a potential association of continuous exposure to trauma with health outcomes for diabetic patients and probably for patients with other chronic diseases. This chronic exposure and the prospects for acute peaks of tension, may lead to allostatic load, and should be monitored for its potential effects on glycemic control and other implications of chronic diseases control and management in the long run. In addition to individual-level interventions, group stress management programs are another effective tool in a “real-world” setting to achieve clinically significant benefits for patients with type 2 diabetes [15], calling for a multidisciplinary diabetes team approach. Health care providers should actually consider residence in a region exposed to continuous terror-related threats as a risk marker requiring special attention and resources.

## Abbreviations

HbA_1c_: glycated haemoglobin
BMI: Body Mass Index
LDL: low-density lipoprotein
GLM: General Linear Models
HMO: Health Maintenance Organization
